# Phyllodes-predominant prostatic stromal sarcoma concurrent with prostate adenocarcinoma: a case report and literature review

**DOI:** 10.3389/fonc.2026.1742518

**Published:** 2026-02-11

**Authors:** Hongguang Lian, Wenfeng Feng, Ming Zhang, Yanhua Tian, Wenxin Wu, Jinfeng Cui, Yuan Wang

**Affiliations:** 1 Department of Pathology, The Second Hospital, Hebei Medical University, Shijiazhuang, China; 2 Department of Imaging, The Second Hospital, Hebei Medical University, Shijiazhuang, China; 3 Department of Urology, The Second Hospital, Hebei Medical University, Shijiazhuang, China; 4 Second Department of Oncology, The Second Hospital, Hebei Medical University, Shijiazhuang, China

**Keywords:** case report, phyllodes-predominant structure, prostate, prostate adenocarcinoma, stromal sarcoma

## Abstract

Prostatic stromal sarcoma (PSS) is a rare malignant mesenchymal tumor originating from the specialized stroma of the prostate. It is a heterogeneous tumor, in which a predominantly phyllodes architecture has been documented only in isolated case reports. The co-occurrence of a phyllodes-predominant PSS and prostate adenocarcinoma (PCA) is even more exceptional. Here, we reported a case of a 52-year-old man with the chief complaint of dysuria for 2 months in 2018. Magnetic resonance imaging (MRI) revealed a mass lesion in the left lobe of the prostate. The patient underwent surgical resection, and the pathological examination revealed PSS with a phyllodes-predominant structure accompanied by PCA. We report this rare case to provide valuable clinical information for clinicians and pathologists and to improve the awareness and diagnostic level of this disease.

## Introduction

Prostatic stromal sarcoma (PSS) is a rare malignant mesenchymal tumor arising from prostate-specific stroma, accounting for less than 0.1% of all malignant prostate neoplasms (1). Marked by significant histological heterogeneity, it presents considerable diagnostic challenges in clinical practice. These challenges stem not only from its morphological overlap with other sarcoma subtypes—which often leads to diagnostic delay or error—but also from the exceptionally low clinical recognition of its even rarer phyllodes-predominant variant, characterized by distinctive leaf-like structures (2, 3).

Notably, the concurrent occurrence of PSS with prostate adenocarcinoma (PCA) represents an even more exceptional clinical scenario, which was described in only a few studies (4, 5). These reported cases lacked phyllodes structures in the PSS component. Herein, we present the first reported case of phyllodes-predominant PSS combined with PCA, detailing the patient’s diagnostic workup, treatment strategy, and 7-year follow-up outcomes. This unique pathological combination exacerbates the complexity of histopathological diagnosis and poses significant challenges for treatment planning and prognostic evaluation, primarily because of the paucity of clinical experience and established guidelines for this rare entity.

## Case presentation

**Figure 1:**
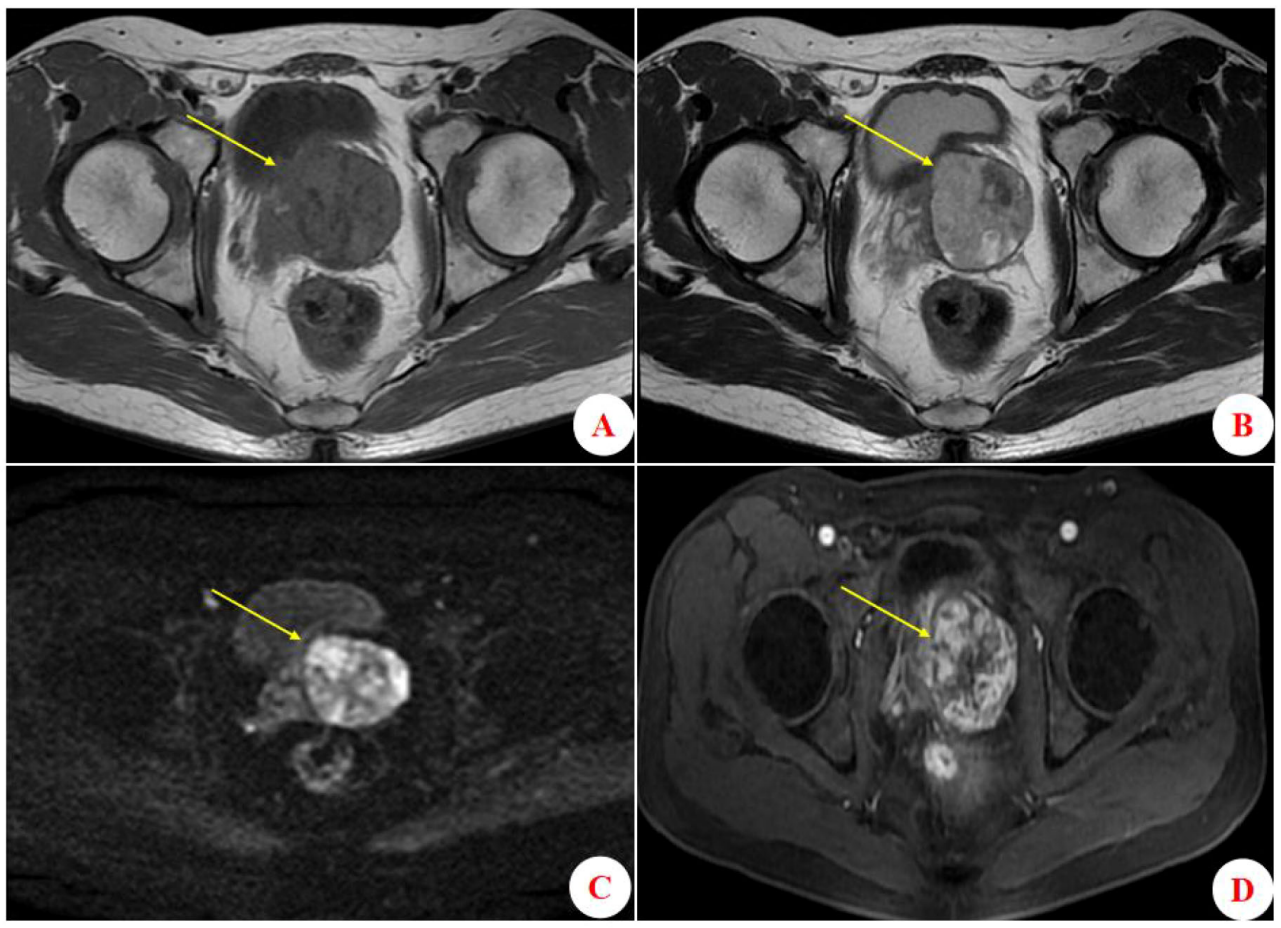
Results of prostate MRI examination. **(A)** Axial T1-weighted imaging (T1WI) showing a hypointense mass lesion (approximately 5.20 × 5.01 × 4.64 cm) in the left prostate. **(B)** Axial T2-weighted imaging (T2WI) shows that the lesion is hyperintense. **(C)** Diffusion-weighted imaging (DWI) reveals diffusional restriction throughout the lesion. **(D)** Axial contrast-enhanced T2WI shows obvious and persistent enhancement of the lesion, without evidence of marginal invasion.

A 52-year-old male patient was admitted to the hospital in November 2018 presenting with symptoms of straining to urinate, accompanied by pollakiuria, nocturia, and urgency of urination. He did not experience dysuria, gross hematuria, pyuria, abdominal distension, or pain. Laboratory tests showed a serum total prostate-specific antigen (TPSA) level of 5.10 ng/mL, with a free PSA (FPSA)/TPSA ratio of 3.73%. Magnetic resonance imaging (MRI) of the urinary system revealed a space-occupying lesion of the prostate (**Figure 1**). A transrectal ultrasound-guided prostate biopsy was performed, and the pathological diagnosis was a prostatic phyllodes tumor. Subsequently, the patient underwent laparoscopic radical prostatectomy. During the procedure, slight adhesions between the posterior wall of the prostate and rectal anterior wall of the rectum were noted; however, the surgery was uneventful, with no evidence of obvious tumor invasion.

**Figure 2:**
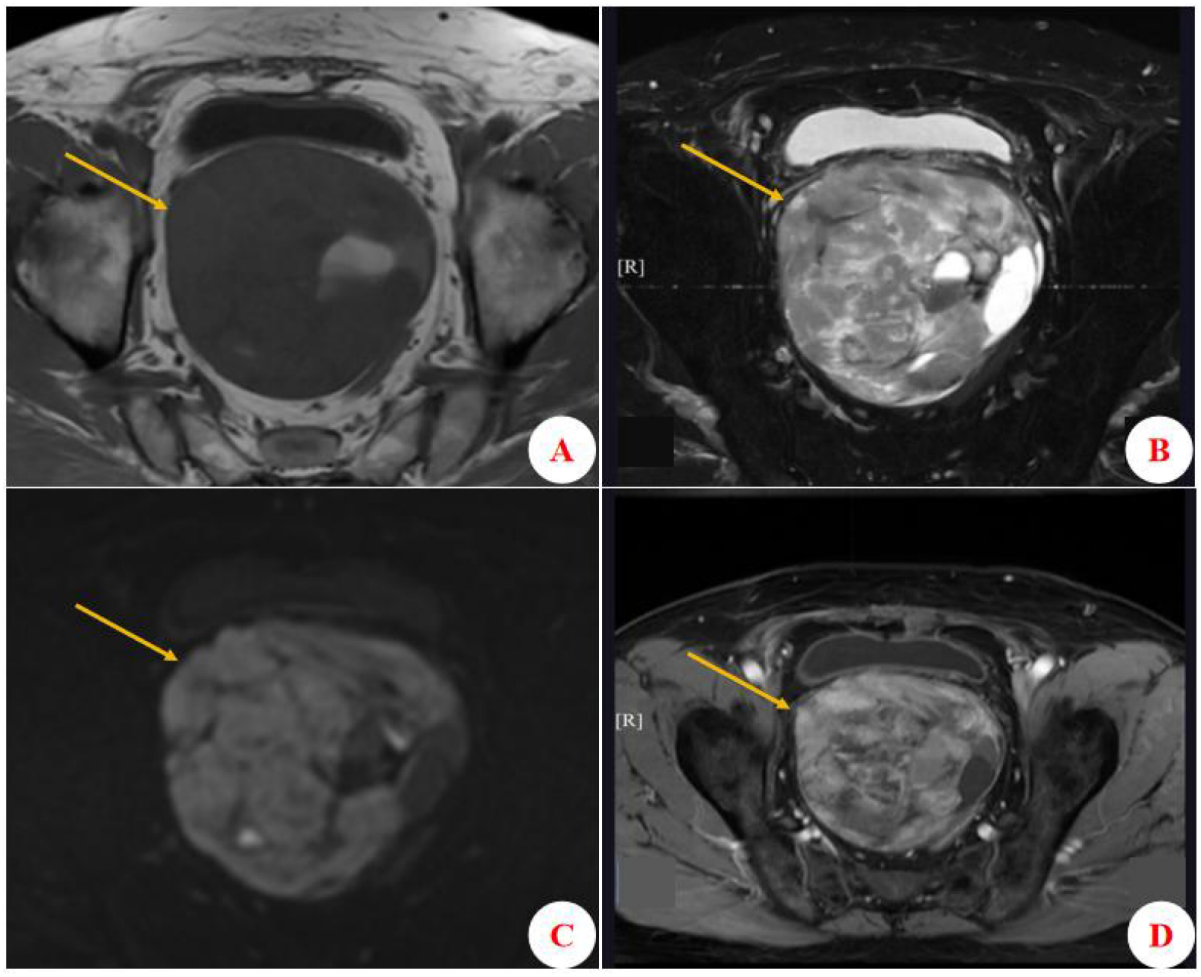
Pelvic MRI at recurrence (7 years postoperative). **(A)** Axial T1-weighted imaging (T1WI) shows a large pelvic mass (approximately 14.80 × 10.20 × 9.40 cm), with hypointense signal. **(B)** Axial T2-weighted imaging (T2WI) demonstrates a large heterogeneous signal mass. The lesion is predominantly solid with multiple cystic areas. **(C)** Diffusion-weighted imaging (DWI) reveals diffusional restriction within the mass. **(D)** Axial contrast-enhanced T2WI reveals marked compression of the bladder and the rectosigmoid junction by the enhancing mass.

The final diagnosis was phyllodes-predominant PSS concurrent with PCA, with the adenocarcinoma component having a Gleason score of 3 + 3 = 6. No definite intravascular tumor thrombus was observed. All surgical resection margins (apex, base, bilateral vas deferens, and seminal vesicles) were negative for tumor. Histological examination of 27 lymph nodes sampled from bilateral iliac vessel regions showed no evidence of metastasis. The patient was followed for 7 years. The first follow-up at 3 weeks postoperatively revealed only mild urinary incontinence. A year later, the follow-up exam showed no tumor recurrence, but the patient subsequently discontinued regular surveillance. In 2025, he was readmitted due to dysuria; MRI (**Figure 2**) confirmed local tumor recurrence, and positron emission tomography–computed tomography (PET–CT; imaging data unavailable) demonstrated multiple metastases, including to the lung and bone. Notably, his serum TPSA level remained normal (0.02 ng/mL) at that time. Given the rapid tumor progression in the setting of a normal PSA level, the recurrence was considered to be predominantly sarcomatous. Therefore, based on soft-tissue sarcoma treatment protocols, a chemotherapeutic regimen of Epirubicin and Ifosfamide with the protective agent Mesna was initiated.

## Pathological findings (initial onset)

**Figure 3:**
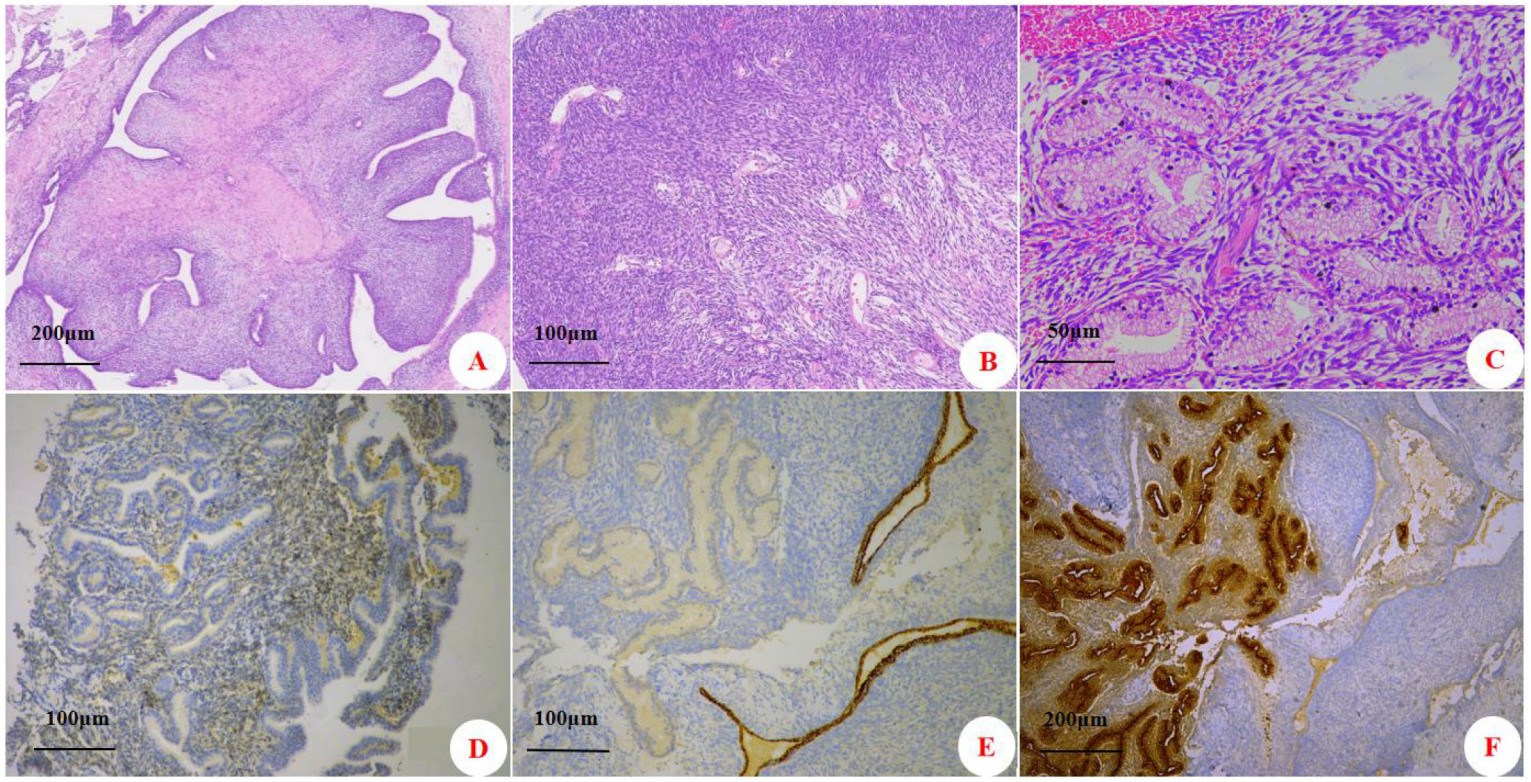
Histopathological and immunohistochemical features of prostatic stromal sarcoma and adenocarcinoma. **(A)** Phyllodes (leaf-like) architecture of the stromal sarcoma (H&E, ×50). **(B)** Hypercellular spindle cell stroma (H&E, ×100). **(C)** Tumor cells arranged in a glandular pattern (H&E, ×200). **(D)** Stromal sarcoma component showing focal weak positivity for progesterone receptor (PR) (IHC, ×100). **(E)** Adenocarcinoma component demonstrating loss of the basal cell marker CK34βE12 (IHC, ×100). **(F)** Adenocarcinoma component showing positive staining for P504S (IHC, ×50).

A nodule with ill-defined margins was identified within the prostate gland. The tumor was predominantly composed of phyllodes structures by glandular epithelium and sarcomatous stroma (**Figure 3A**). The epithelial cells showed no obvious atypia. The stromal component was abundant and consisted of spindle-shaped cells (**Figure 3B**). These stromal cell nuclei exhibited mild to moderate atypia, with a mitotic count of approximately 7 per 10 high-power fields (HPF). No hemorrhage or necrosis was observed. Additionally, a 0.3 × 0.2 cm focus of PCA was identified in the prostatic tissue adjacent to the phyllodes tumor (**Figure 3C**). Immunohistochemical (IHC) analysis showed that the PSS component was diffusely positive for Vimentin and focally positive for CD34, ER, and PR (**Figure 3D**); its Ki-67 proliferation index was 15%. At the same time, the PCA component was negative for the basal cell markers CK34βE12 (**Figure 3E**) and P63, while it was positive for P504S (**Figure 3F**) and PSA.

## Discussion

Prostatic phyllodes tumor is a rare mesenchymal neoplasm (6) with unique clinical and pathological features, historically described under various names (7). In 2004, WHO classified it as prostatic-specific stromal tumors (8), which were further subclassified into PSS and stromal tumor of uncertain malignant potential (STUMP). This distinction is based on histopathological criteria including the extent of mesenchymal infiltration, mitotic count, and the degree of mesenchymal overgrowth (9, 10). Histologically, PSS exhibits diffuse stromal proliferation with various patterns, such as storiform, epithelioid, fibrosarcomatous, phyllodes tumor, or no specific pattern. Phyllodes-predominant PSS is composed of both epithelial and stromal components. The epithelial component is typically compressed by the stroma, resulting in characteristic leaf-like (phylloded) architectures. The stromal component is characterized by diffuse spindle cell proliferation, increased cellularity, cytological atypia, frequent mitotic figures, and often necrosis. Immunohistochemically, the stromal cells may express CD34 and PR. In the present case, a biopsy initially indicated a phyllodes tumor, prompting laparoscopic radical prostatectomy. Histopathological examination of the resected specimen revealed a phyllodes-predominant architecture. The stromal component showed high cellular density, mild to moderate nuclear atypia, an elevated mitotic index (approximately 7 per 10 HPF), and immunoreactivity for CD34 and PR. Notably, no definitive necrosis was identified. Based on these features, a final diagnosis of phyllodes-predominant PSS was rendered.

PSA levels in patients with PSS are typically within the normal range (11). However, in this patient, the TPSA was mildly elevated (5.10 ng/mL) and the FPSA/TPSA ratio was significantly low, raising suspicion for concurrent prostate cancer. Careful histopathological examination, supported by IHC findings, subsequently confirmed a small (0.3 × 0.2 cm) focus of PCA in the prostatic tissue adjacent to the PSS.

The co-occurrence of PSS with PCA is exceptionally rare, and the underlying pathogenic mechanism remains unclear. Previous studies have established the critical role of epithelial–stromal interactions in the development, progression and metastasis of prostate cancer (12–16). For instance, Elo et al. (17) demonstrated that “stromal activation promotes the development of prostatic intraepithelial neoplasia (mPIN) lesions and carcinoma in transgenic mice.” In this case, the prominent phyllodes structures of the PSS provided an abnormally extensive epithelial–stromal interface. Whether this distinctive morphological architecture plays a specific role in modulating the local microenvironment to initiate or facilitate concurrent PCA warrants further investigation.

PSS is characterized by rapid progression and a poor prognosis, with common metastatic sites including bone, liver, and lung (18, 19). Because of its rarity, large-scale clinical studies are lacking, and no standardized treatment regimen has been established. For cases involving both sarcoma and adenocarcinoma, radical surgical resection is considered the primary treatment. Multimodal therapy combining surgery with adjuvant treatment may improve clinical outcomes. When metastasis to bones, lymph nodes, or other organs are detected, meticulous histological examination of the metastatic foci is crucial to identify whether the metastasis originates from the sarcoma or the adenocarcinoma component, as misidentification can lead to inappropriate therapy and adversely impact survival. In the present case, the patient developed extensive metastases (to lung, pelvic cavity, and bone) 7 years postoperatively. Regrettably, a biopsy of the metastatic lesions was not performed due to concerns about potential tumor dissemination. Thus, the dominant component at recurrence was unclear. Notably, at the time of recurrence, the serum TPSA level—a reliable biomarker for PCA surveillance—remained normal (0.02 ng/mL). Furthermore, considering that the PSS component typically exhibits more aggressive behavior than PCA, the recurrent disease was clinically presumed to be predominantly sarcomatous. Consequently, a chemotherapy regimen based on soft-tissue sarcoma protocols was initiated.

This case highlights the possibility of concurrent PSS and adenocarcinoma (PCA). Thus, thorough pathological examination of resection specimens is crucial to exclude a coexisting PCA, especially in patients with elevated serum PSA levels. Furthermore, a rigorous, long-term follow-up regimen is necessary to monitor for recurrence and to determine the origin (sarcomatous vs. carcinomatous) of any recurrent disease, which is critical for guiding subsequent therapy.

## Data Availability

The original contributions presented in the study are included in the article/supplementary material. Further inquiries can be directed to the corresponding author.
